# Supplementary material for: Effect of community-based newborn care implementation strategies on access to and effective coverage of possible serious bacterial infection (PSBI) treatment for sick young infants during COVID-19 pandemic
Source: PLoS One. 2024 Mar 25;19(3):e0300880. doi: 10.1371/journal.pone.0300880 (PMC10962833; doi:10.1371/journal.pone.0300880)
Supplement: Attachment — Submitted filename: PONE-D-23-12889_reviewer- PSBI Ethiopia with comments.pdf [file pone.0300880.s004.pdf]

PLOS ONE

Effect of community-based newborn care implementation strategies on access to and effective coverage of possible serious bacterial infection (PSBI) treatment for sick young infants during COVID-19 pandemic

--Manuscript Draft--

**Manuscript Number:**	PONE-D-23-12889
**Article Type:**	Research Article
**Full Title:**	Effect of community-based newborn care implementation strategies on access to and effective coverage of possible serious bacterial infection (PSBI) treatment for sick young infants during COVID-19 pandemic
**Short Title:**	Access to and effective coverage of possible serious bacterial infection treatment for sick young infants
**Corresponding Author:**	Gizachew Tadele Tiruneh, Ph.D.<br>JSI Research and Training Institute Inc<br>Adddis Ababa, ETHIOPIA
**Keywords:**	community-based management of common childhood illnesses; Ethiopia; effective coverage; implementation science research; newborn infection; neonatal sepsis; possible serious bacterial infection (PSBI)
**Abstract:**	Background<br><br>In Ethiopia, neonatal mortality is persistently high. The country has been implementing community-based treatment of possible serious bacterial infection (PSBI) in young infants when referral to a hospital is not feasible since 2012. However, access to and quality of PSBI services remained low and were worsened by COVID-19. From November 2020 to June 2022, we conducted implementation research to mitigate the impact of COVID-19 and improve PSBI management implementation uptake and delivery in two woredas in Ethiopia.<br><br>Methods<br><br>In April-May 2021, guided by implementation research frameworks, we conducted formative research to understand the PSBI management implementation challenges, including those due to the COVID-19 pandemic. Through a participatory process engaging stakeholders, we designed adaptive implementation strategies to bridge identified gaps using mechanism mapping to achieve implementation outcomes. Strategies included training and coaching, supportive supervision and mentorship, technical support units, improved supply of essential commodities, and community awareness creation about PSBI and COVID-19. We conducted cross-sectional household surveys in the two woredas before (April 2021) and after the implementation of strategies (June 2022) to measure changes in targeted outcomes.<br><br>Results<br><br>We interviewed 4,262 and 4,082 women who gave live birth 2-14 months before data collection and identified 374 and 264 PSBI cases in April 2021 and June 2022, respectively. The prevalence of PSBI significantly decreased (p-value = 0.018) from 8.7% in April 2021 to 6.4% while the mothers' care-seeking behavior from medical care for their sick newborns increased significantly from 56% to 91% (p-value <0.01). Effective coverage of severely ill young infants that took appropriate antibiotics significantly improved from 33% [95% CI: 25.5-40.7] to 62% [95% CI: 51.0-71.6]. Despite improvements in the uptake of PSBI treatment, persisting challenges at the facility and systems levels impeded optimal PSBI service delivery and uptake, including perceived low quality of service, lack of community trust, and shortage of supplies.<br><br>Conclusion<br><br>The participatory design and implementation of adaptive COVID-19 strategies effectively improved the uptake and delivery of PSBI treatment. Support systems were

	critical for frontline health workers to deliver PSBI services and create a resilient community health system to provide quality PSBI care during the pandemic. Additional strategies are needed to address persistent gaps, including improvement in client-provider interactions, supply of essential drugs, and increased social mobilization strategies targeting families and communities to further increase uptake.
Order of Authors:	Gizachew Tadele Tiruneh, Ph.D.
	Nebreed Fesseha
	Dessalew Emaway
	Wuleta Betemariam
	Tsinuel Girma Nigatu
	Hema Magge
	Lisa Ruth Hirschhorn
Additional Information:	
**Question**	**Response**
**Financial Disclosure**<br><br>Enter a financial disclosure statement that describes the sources of funding for the work included in this submission. Review the submission guidelines for detailed requirements. View published research articles from *PLOS ONE* for specific examples.<br><br>This statement is required for submission and **will appear in the published article** if the submission is accepted. Please make sure it is accurate.<br><br>**Unfunded studies**<br>Enter: *The author(s) received no specific funding for this work.*<br><br>**Funded studies**<br>Enter a statement with the following details:<br>• Initials of the authors who received each award<br>• Grant numbers awarded to each author<br>• The full name of each funder<br>• URL of each funder website<br>• Did the sponsors or funders play any role in the study design, data collection and analysis, decision to publish, or preparation of the manuscript?<br>• **NO** - Include this sentence at the end of your statement: *The funders had no role in study design, data collection and analysis, decision to publish, or preparation of the manuscript.*<br>• **YES** - Specify the role(s) played.	The article write-up and publication fee were supported by the Bill & Melinda Gates Foundation, Investment Number INV-024241. JSI Research & Training Institute, Inc. has helped us in the form of salaries for authors [GT, NF, DE, and WB]. However, none of the funders played a role in the study design, data collection, analysis, decision to publish, or manuscript preparation.

* typeset	
**Competing Interests**<br><br>Use the instructions below to enter a competing interest statement for this submission. On behalf of all authors, disclose any competing interests that could be perceived to bias this work—acknowledging all financial support and any other relevant financial or non-financial competing interests.<br><br>This statement is required for submission and **will appear in the published article** if the submission is accepted. Please make sure it is accurate and that any funding sources listed in your Funding Information later in the submission form are also declared in your Financial Disclosure statement.<br><br>View published research articles from *PLOS ONE* for specific examples.<br><br>**NO authors have competing interests**<br><br>Enter: *The authors have declared that no competing interests exist.*<br><br>**Authors with competing interests**<br><br>Enter competing interest details beginning with this statement:<br><br>*I have read the journal's policy and the authors of this manuscript have the following competing interests: [insert competing interests here]*<br><br>* typeset	The authors declare that they have no competing interests. The authors [GT, NF, DE, and WB] work for JSI Research & Training Institute, Inc., a commercial company. We declare that this commercial affiliation does not alter our adherence to the journal's policies on sharing data and materials. One of the authors of this manuscript (HM) works for the Gates Foundation. We would like to declare that we do not have any conflict of interest with the Gates Foundation-paid staff in preparing this manuscript.
**Ethics Statement**<br><br>Enter an ethics statement for this submission. This statement is required if the study involved:	Ethical clearance was obtained from the Ethiopian Public Health Association (EPHA) Research Ethics Review Committee (Reference #: EPHA/OG/166/21 dated April 16, 2021) and renewed for the end-line survey (Reference #: EPHA/OG/781/22 dated April 18, 2022). Written permission to undertake the study was sought from the zonal health department and institutions. Informed verbal consent and voluntary participation were ensured. All study participants were informed about the study’s purpose, benefits, and

• Human participants<br>• Human specimens or tissue<br>• Vertebrate animals or cephalopods<br>• Vertebrate embryos or tissues<br>• Field research<br><br>Write "N/A" if the submission does not require an ethics statement.<br><br>General guidance is provided below. Consult the submission guidelines for detailed instructions. **Make sure that all information entered here is included in the Methods section of the manuscript.**	hazards, including their right to opt out and to respond to questions without consequence. If the respondent was less than 18 years old, then consent was sought from her husband or guardian. The authors confirm that all methods were carried out in accordance with the relevant guidelines and regulations.

Powered by Editorial Manager® and ProduXion Manager® from Aries Systems Corporation

**Format for specific study types**<br><br>**Human Subject Research (involving human participants and/or tissue)**<br>• Give the name of the institutional review board or ethics committee that approved the study<br>• Include the approval number and/or a statement indicating approval of this research<br>• Indicate the form of consent obtained (written/oral) or the reason that consent was not obtained (e.g. the data were analyzed anonymously)<br><br>**Animal Research (involving vertebrate animals, embryos or tissues)**<br>• Provide the name of the Institutional Animal Care and Use Committee (IACUC) or other relevant ethics board that reviewed the study protocol, and indicate whether they approved this research or granted a formal waiver of ethical approval<br>• Include an approval number if one was obtained<br>• If the study involved *non-human primates*, add *additional details* about animal welfare and steps taken to ameliorate suffering<br>• If anesthesia, euthanasia, or any kind of animal sacrifice is part of the study, include briefly which substances and/or methods were applied<br><br>**Field Research**<br><br>Include the following details if this study involves the collection of plant, animal, or other materials from a natural setting:<br>• Field permit number<br>• Name of the institution or relevant body that granted permission	
**Data Availability**<br><br>Authors are required to make all data underlying the findings described fully available, without restriction, and from the time of publication. PLOS allows rare exceptions to address legal and ethical concerns. See the PLOS Data Policy and FAQ for detailed information.	Yes - all data are fully available without restriction

Powered by Editorial Manager® and ProduXion Manager® from Aries Systems Corporation

A Data Availability Statement describing where the data can be found is required at submission. Your answers to this question constitute the Data Availability Statement and **will be published in the article**, if accepted.<br><br>**Important:** Stating 'data available on request from the author' is not sufficient. If your data are only available upon request, select 'No' for the first question and explain your exceptional situation in the text box.<br><br>Do the authors confirm that all data underlying the findings described in their manuscript are fully available without restriction?	
**Describe where the data may be found in full sentences. If you are copying our sample text, replace any instances of XXX with the appropriate details.**<br><br>• If the data are **held or will be held in a public repository**, include URLs, accession numbers or DOIs. If this information will only be available after acceptance, indicate this by ticking the box below. For example: *All XXX files are available from the XXX database (accession number(s) XXX, XXX.)*.<br>• If the data are all contained **within the manuscript and/or Supporting Information files**, enter the following: *All relevant data are within the manuscript and its Supporting Information files.*<br>• If neither of these applies but you are able to provide **details of access elsewhere**, with or without limitations, please do so. For example:<br><br>*Data cannot be shared publicly because of [XXX]. Data are available from the XXX Institutional Data Access / Ethics Committee (contact via XXX) for researchers who meet the criteria for access to confidential data.*<br><br>*The data underlying the results presented in the study are available from (include the name of the third party*	The dataset used and analyzed during this study is included as supplementary information to this article (Additional file 3).

and contact information or URL).<br>• This text is appropriate if the data are owned by a third party and authors do not have permission to share the data.<br><br>* typeset	
Additional data availability information:	

Powered by Editorial Manager® and ProduXion Manager® from Aries Systems Corporation

Effect of community-based newborn care implementation strategies on access to and effective coverage of possible serious bacterial infection (PSBI) treatment for sick young infants during COVID-19 pandemic

Gizachew Tadele Tiruneh[1]*, Nebreed Fesseha[1], Dessalew Emaway[1], Wuleta Betemariam[2], Tsinuel Girma Nigatu[3], Hema Magge[4], and Lisa Ruth Hirschhorn[5]

[1] The Last Ten Kilometers (L10K) Project, JSI Research & Training Institute, Inc., Addis Ababa, Ethiopia

[2] John Snow Inc., Washington DC, USA

[3] Department of Pediatrics and Child Health, Jimma University, Ethiopia and Fenot Project - School of Population and Public Health, University of British Columbia, Canada

[4] Bill & Melinda Gates Foundation, Seattle, Washington, USA

[5] Feinberg School of Medicine and Havey Institute of Global Health, Northwestern University, Chicago, IL, USA

*Correspondence: gizt121@gmail.com

Email addresses of authors: gizt121@gmail.com (GT); nebreed_fesseha@et.jsi.com (NF); dessalew_emaway@et.jsi.com (DE); wuleta_betemariam@jsi.com (WB); Tsinuel.nigatu@ubc.ca (TG), Hema.Magge@gatesfoundation.org (HM); and lisa.hirschhorn@northwestern.edu (LH)

Abstract

Background: In Ethiopia, neonatal mortality is persistently high. The country has been implementing community-based treatment of possible serious bacterial infection (PSBI) in young infants when referral to a hospital is not feasible since 2012. However, access to and quality of PSBI services remained low and were worsened by COVID-19. From November 2020 to June 2022, we conducted implementation research to mitigate the impact of COVID-19 and improve PSBI management implementation uptake and delivery in two woredas in Ethiopia.

Methods: In April-May 2021, guided by implementation research frameworks, we conducted formative research to understand the PSBI management implementation challenges, including those due to the COVID-19 pandemic. Through a participatory process engaging stakeholders, we designed adaptive implementation strategies to bridge identified gaps using mechanism mapping to achieve implementation outcomes. Strategies included training and coaching, supportive supervision and mentorship, technical support units, improved supply of essential commodities, and community awareness creation about PSBI and COVID-19. We conducted cross-sectional household surveys in the two woredas before (April 2021) and after the implementation of strategies (June 2022) to measure changes in targeted outcomes.

Results: We interviewed 4,262 and 4,082 women who gave live birth 2-14 months before data collection and identified 374 and 264 PSBI cases in April 2021 and June 2022, respectively. The prevalence of PSBI significantly decreased (p-value = 0.018) from 8.7% in April 2021 to 6.4% while the mothers' care-seeking behavior from medical care for their sick newborns increased significantly from 56% to 91% (p-value <0.01). Effective coverage of severely ill young infants that took appropriate antibiotics significantly improved from 33% [95% CI: 25.5-40.7] to 62% [95% CI:

51.0-71.6]. Despite improvements in the uptake of PSBI treatment, persisting challenges at the facility and systems levels impeded optimal PSBI service delivery and uptake, including perceived low quality of service, lack of community trust, and shortage of supplies.

Conclusion: The participatory design and implementation of adaptive COVID-19 strategies effectively improved the uptake and delivery of PSBI treatment. Support systems were critical for frontline health workers to deliver PSBI services and create a resilient community health system to provide quality PSBI care during the pandemic. Additional strategies are needed to address persistent gaps, including improvement in client-provider interactions, supply of essential drugs, and increased social mobilization strategies targeting families and communities to further increase uptake.

Keywords: community-based management of common childhood illnesses, Ethiopia, effective coverage, implementation science research, newborn infection, neonatal sepsis, possible serious bacterial infection (PSBI)

Background

Sub-Saharan Africa had the world's highest neonatal mortality rate (NMR) at 27 deaths per 1,000 live births in 2021 [1]. In Ethiopia, the NMR is a major public health problem that has not significantly declined since 2000, and currently stands at 33 per 1,000 live births, contributing to about 56% of under-five child mortality [2]. Considering the slow deceleration rate of NMR and the impact of COVID-19, most sub-Saharan African countries, including Ethiopia, are at risk of missing the Sustainable Development Goal (SDG) target for neonatal mortality [1].

Severe neonatal infections are among the leading causes of death accounting for half a million newborn deaths in 2019 [3]. In resource-limited settings, access to inpatient treatment is difficult for many young infants with signs of severe infection due to limited accessibility to acceptable or affordable care [4, 5]. Informed by evidence from low-and-middle-income countries (LMICs) that demonstrated the effectiveness of simplified regimens, including injectable and oral antibiotics, the World Health Organization (WHO) recommends the management of possible serious bacterial infection (PSBI) outside of the hospital setting when referral is not feasible [6].

Ethiopia adopted community-based newborn infection management in 2012 and incorporated it within the integrated community-based management (iCCM) of common childhood illnesses platform to improve the use of iCCM services for newborns and young infants by Health Extension Workers (HEWs) when referral is not feasible [7]. However, the crude and effective coverage of PSBI management remains low. The COVID-19 pandemic [8, 9] and multiple pre-existing contextual factors resulted in increased barriers to uptake and delivery. These included erratic supply of essential drugs and poor logistics management systems [10, 11]; suboptimal supportive

and referral links [10-12]; suboptimal quality of care [13]; community misperceptions about newborn illnesses [14, 15]; inaccessibility of facilities; and socio-cultural beliefs [10, 15] which all affected the implementation and uptake of iCCM services including PSBI diagnosis and treatment [16].

Improving both access to and quality of PSBI management is needed to address barriers to effective improvement of newborn health outcomes. However, expanding access and delivery without focusing on quality is not sufficient to achieve the intended outcomes. Evidence in LMICs showed that effective quality coverage is considerably lower than coverage for maternal and child health services ignoring quality [17]. As such, the global health community recognized high-quality health care as an important component of efforts to reach SDG 3 for the achievement of universal health coverage [18, 19]. Monitoring trends in effective coverage of PSBI management and place of treatment (health facility or community) is crucial to assess the quality of community-based iCCM programs. However, in Ethiopia, population-level data on the effective coverage of PSBI treatment and place of treatment are scarce.

Between November 2020 and June 2022, the Bill & Melinda Gates Foundation (BMGF) funded the JSI Research & Training Institute Inc. (JSI) Last Ten Kilometers (L10K) Project to conduct implementation research (IR) in Ethiopia to design and support strengthening the adaptive implementation of community-based management of PSBI for sick young infants and to mitigate the impact of COVID-19 on neonatal mortality. Employing an implementation science evaluation framework, we evaluated the effects of these health systems and community strategies on

community-based neonatal sepsis management on access to and effective coverage of PSBI for sick young infants.

Methods

Study context and settings

Ethiopia's primary-level care system includes a primary hospital, health centers, and health posts; supported by secondary and tertiary-level care. The primary health system in a typical woreda (i.e., district) should have one primary hospital, 4-5 health centers, 20-25 health posts, 40-50 Health Extension Workers (HEWs), and about 4,000 Women Development Army (WDA) volunteers to serve a population of approximately 100,000 people [20]. Primary health care services include promotive, preventative, curative, and rehabilitative services at facilities (i.e., at health posts, health centers, and primary hospitals), at home or in the community, outreach services through the Health Extension Program (HEP), and the engagement of community volunteers. Health posts are staffed with two female HEWs and supported by a network of WDAs providing promotive maternal and newborn services and some basic antepartum and postpartum counseling and referral services.

This implementation research was conducted at Dembecha and Lume woredas of the Amhara and Oromia regions. The two woredas are comprised of 11 health centers and 66 health posts that serve an estimated 250,000 rural people.

Participatory design of strategies and pathways of adaptive implementation

We employed IR frameworks in designing, implementing, and evaluating the adaptive implementation of PSBI treatment during COVID-19 in Ethiopia. The Reach, Effectiveness, Adoption, Implementation, and Maintenance (RE-AIM) [21] and Consolidated Framework for Implementation Research (CFIR) [22] frameworks were used to define outcomes and identify factors that needed to be addressed. Figure 1 below depicts the simplified process of the implementation research to explore the different dimensions of RE-AIM.

Figure 1: Program design and implementation process, 2021

In April 2021, we conducted a narrative review of existing literature followed by formative research to identify implementation challenges. Details of the findings are presented elsewhere [16]. In May 2021, we facilitated a participatory design of strategies by engaging relevant stakeholders to systematically develop, identify, categorize, and prioritize implementation strategies to address identified and newly emerged barriers. Then, we matched our implementation strategies with the Effective Practice and Organization of Care (EPOC) [23] and Expert Recommendations for Implementing Change (ERIC) [24] taxonomies and strategies identified from previous implementation research on the management of PSBI when referral was not feasible in Ethiopia, Malawi, Nigeria, Bangladesh, Pakistan, and India at the individual, community, facility, and system levels [4, 12, 25-32]. At the individual level, we identified on-site coaching and training of HEWs by PSBI/iCCM-trained focal mentors from health centers as strategies. At the facility and systems-level, we identified the following strategies: coordinated referrals between health posts and health centers; the establishment of a technical support unit

to facilitate implementation across the area; strengthening of the supply chain, support system, and links; integration of management of PSBI implementation in the woreda health system work stream; and integration of COVID-19 and routine services. Details of the implementation challenges, strategies, mechanisms of action, and their outcomes can be found elsewhere [16].

Evaluation design

We evaluated the effects of the implementation strategies on access to and treatment cascade of PSBI using before-after household surveys. Data were collected from 18 April-24 May 2021 for the baseline and from 06 June-09 July 2022 for the end-line. In addition, at the end-line, a programmatic qualitative study was conducted to explore the perceived impact of the strategies and barriers to implementation.

Sample size and sampling technique

With the assumption of 3% estimated live births, 5% prevalence of PSBI, and 56% of mothers seeking medical care [33], we estimated that 375 neonatal sepsis cases and 210 cases who sought medical care could be obtained in the study woredas. This was based on an estimated population of about 250,000 people and 50,000 households. The adequacy of the sample size for the household survey was determined as sufficient using a double population formula in a comparative before and after cross-sectional study design. This would be sufficiently powered to detect a 20-percentage point change in sick newborn care-seeking due to the intervention. The assumption to calculate the sample size considered that 56% of mothers sought medical care for ill babies in the neonatal period [33], 95% confidence level ($Z\alpha/2=1.96$), power of 80%, and

Line 156, P. 9: Please explain what are kebeles. Not discussed earlier.

design effect of 1.5. Accordingly, all 66 kebeles were included in the study, and a cohort of mothers with infants 2-14 months was identified and interviewed.

We recruited all eligible households after obtaining an updated list of households with under-15-month infants using unique household identifiers from the Community Health Information System (CHIS) from all 66 health posts. These records are available at health posts and shelved in numeric order. When a complete list of households was not available at health posts, an ad hoc list of households was established by data collectors with the help of HEWs and community volunteers before data collection. Accordingly, 4,262 and 4,082 mothers with infants 2-14 months were identified and interviewed during baseline and end-line, respectively. For the qualitative component of the study, the theoretical sampling technique was used to collect information until saturation in each data category was achieved.

Data collection

We conducted before-after cross-sectional household surveys of women who gave live birth 2-14 months before data collection and resided in the two woredas. Survey data included household and sociodemographic characteristics, experiences related to using maternal and newborn health services, care-seeking for sick infants and children, and knowledge and risk perception of COVID-19. We used a structured questionnaire translated into Amharic and Oromiffa local languages using a web-based SurveyCTO mHealth platform on data collector smartphones (Additional file 1).

During the end line, we conducted 41 in-depth interviews (IDIs) with 19 HEWs, 11 health center directors, nine health center iCCM focal persons, and two woreda child health officers using an

open-ended questionnaire-guided interview that was audio-recorded (Additional file 2). Interviews were conducted in the Amharic and Oromiffa languages by experienced qualitative research consultants.

Outcome measures

In this paper, effective coverage of PSBI treatment is defined as coverage of PSBI treatment adjusted for receipt of the appropriate antibiotics (gentamicin injection and/or amoxicillin) among young infants 0-59 days with signs and symptoms of severe bacterial infection (e.g., difficulty breathing, chest in-drawing, ceasing to feed well, unusually hot or cold, less active than usual, and/or convulsions) who sought care from the appropriate provider (either from health professionals or HEWs). First, we calculated the crude service contact coverage as the percent of sick young infants with PSBI symptoms who sought care from appropriate providers. Then, we calculated intervention-adjusted coverage of sick young infants with PSBI who sought care from appropriate providers who received antibiotics treatment. Finally, we calculated the effective coverage cascade, beginning with the target population, crude service contact coverage, and intervention-adjusted coverage adjusting for the recipient of appropriate treatment [34, 35].

Line 191- would be useful to define appropriate rtreatment. Is it 7 days opf antibiotics or patial treatment os also considered appropriate .

Data analysis

Quantitative data analysis was conducted using Stata version 15.1. Socio-demographic data were summarized by frequency tables and summary statistics. The study was adjusted for the cluster survey design effect. We estimated statistically significant (p<.05) differences in the prevalence of PSBI and care-seeking behavior of mothers between the baseline and the end-line surveys using chi-square statistical tests.

The audio recordings from in-depth interviews were transcribed verbatim for the qualitative data, and the transcript texts were exported to ATLAS.ti software for thematic analysis. These findings are integrated with the quantitative component of the evaluation.

Ethics approval and consent to participate

Ethical clearance was obtained from the Ethiopian Public Health Association (EPHA) Research Ethics Review Committee (Reference #: EPHA/OG/166/21 dated April 16, 2021) and renewed for the end-line survey (Reference #: EPHA/OG/781/22 dated April 18, 2022). Written permission to undertake the study was sought from the zonal health department and institutions. Informed verbal consent and voluntary participation were ensured. All study participants were informed about the study's purpose, benefits, and hazards, including their right to opt out and to respond to questions without consequence. If the respondent was less than 18 years old, then consent was sought from her husband or guardian. We deidentified and stored respondents' information. The authors confirm that all methods were carried out in accordance with the relevant guidelines and regulations.

Results

Respondent characteristics

In the household survey, 4,262 and 4,081 women with children aged 2-14 months were interviewed during April 2021 and June 2022, respectively. More than two-thirds of the respondents were in the 20-34 year age group. The majority had no formal education or literacy

skills (56% in April 2021 and 53% in June 2022), and about half of them lived 30 minutes or more walking distance from health posts or health centers (47% in April 2021 and 55% in June 2022).

There were differences in the background characteristics of the respondents for maternal education, number of children, and distance to health facilities. In the end-line survey, respondents were more likely better educated, had less parity, and resided within 30 minutes of the facility (Table 1).

Table 1: Characteristics of study participants during baseline and end-line survey periods, 2022

Background characteristics	Baseline	End-line
**Age of the mother (in years)**		
15-19	211 (5.0)	183 (4.5)
20-34	2,943 (69.0)	2,845 (69.7)
35-49	1,108 (26.0)	1,053 (25.8)
**Maternal education**		
Cannot read/no formal education	2,365 (55.5)	2,147 (52.6)
Primary school completed	853 (20.0)	805 (19.7)
Secondary and higher	1,044 (24.5)	1,129 (27.7)**
**Wealth quintile**		
Most poor	805 (18.9)	880 (21.6)
More poor	827 (19.4)	826 (20.2)
Poor	849 (19.9)	818 (20.1)
Less poor	879 (20.6)	786 (19.3)
Least poor	903 (21.2)	771 (18.9)
**Number of children**		
1	1,043 (24.5)	1,069 (26.2)
2-3	1,565 (36.7)	1,562 (38.3)
4+	1,653 (38.8)	1,450 (35.5)**
**Distance to the nearest health facility (walking distance)**		
< 30 min	1,992 (46.7)	2,247 (55.1)
30 min-1 hr	1,500 (35.2)	1.279 (31.3)
>1 hr	771 (18.81	555 (13.6)*
**Administrative woreda**		
Dembecha	2,494 (58.5)	2,392 (58.6)

Lume	1,768 (41.5)	1,689 (41.4)
**Total number of respondents**	**4,262 (100)**	**4,081 (100)**

** Statistically significant difference (p<0.01) and ** (<0.05) between baseline and end-line surveys*

Changes in prevalence of PSBI and care-seeking behavior

The prevalence of any history of illness during the neonatal period was significantly reduced from 11.6% [95% CI: 9.8-13.7] in April 2021 to 8.4% [95% CI: 6.9-10.3] in June 2022. Similarly, the prevalence of PSBI reduced from 8.4% [95% CI: 7.0-10.0] in April 2021 to 6.2% [95% CI: 4.8-7.8] in June 2022. The care-seeking behavior of mothers for their severely sick neonates had significantly improved from 56.1% [95% CI: 45.3-66.4] in April 2021 to 91.3% [95% CI: 87.9-93.8] in June 2022 (Table 2).

Table 2: Changes in the prevalence of neonatal illness and mother's care-seeking behaviors during baseline and end-line survey periods, June 2022

Variables	Baseline	End-line
**Neonatal illness**		
History of illness	494 (11.6)	345 (8.4)*
Formal health care seeking for ill neonates	294 (57.6)	313 (87.8)*
Number of sick young infants	***510***	***357***
**Neonatal severe infections (PSBI)**		
Newborns with severe neonatal infection	357 (8.4)	250 (6.1)**
Neonatal infection treated with antibiotics	210 (56.1)	241 (91.3)*
Number of PSBI cases	***374***	***264***

** Statistically significant difference (p<0.01) and ** (<0.05) between baseline and end-line surveys*

Change in the place and time of care-seeking and treatment

A statistically significant proportion of mothers sought care for PSBI at health posts after the intervention: 4.2% (95% CI: 2.0-9.0%) at baseline to 26.4 % (95% CI: 16.4-39.5%) at the end-line (Figure 2).

Figure 2: Changes in the place of care-seeking during baseline and end-line survey periods, 2022

Effective PSBI treatment coverage

We analyzed the cascade across the PSBI care pathway from the baseline and end-line surveys. Accordingly, the proportion of infants identified with PSBI that took any drug significantly improved from 52.9% [95% CI: 42.7-62.8] in April 2021 to 82.1% [95% CI: 75.0-87.5] in June 2022. Likewise, the proportion of severely ill young infants that took appropriate antibiotics significantly improved from 32.6% [95% CI: 25.5-40.7] in April 2021 to 61.8% [95% CI: 51.0-71.6] in June 2022 as presented in the error bar (Figure 3).

Figure 3: Changes in PSBI treatment cascade coverage over time, 2022

Perceived effectiveness of the strategies

According to IDI respondents, the implementation of the adaptive strategies was helpful to 1) recover services interrupted due to COVID-19; 2) enhance the capacity of HEWs; 3) improve community awareness about the iCCM/PSBI service availability; and 4) improve the care-seeking behavior of mothers for their sick young infants, which corroborates the quantitative finding.

Respondents mentioned that one of the perceived effects of this IR was a recovery of routine services from the COVID-19 pandemic.

"… the community, at some point, has stopped the service due to COVID-19. But then, we persuaded the community to resume using the service after a temporary halt." (iCCM focal person respondent)

“To our delight, now everything is turning out well or having the intended result, and most community members are using the [iCCM] services at the health posts.” (iCCM Focal Person respondent)

Other effects of this investment mentioned were enhanced capacity of health centers’ strengthened ability to support HEWs:

“The success achieved in boosting the capacity of HEWs is worth mentioning. Generally, the support from JSI was immense in many aspects, including the training provided for our health professionals, which was unthinkable during the COVID-19 era. More importantly, the support we received equipped us with the means to improve the capacity of HEWs.” (iCCM focal person respondent)

Ongoing implementation challenges

Despite improved health post readiness and demand generation activities to improve the uptake and delivery of iCCM/PSBI services, respondents highlighted remaining barriers impeding optimal iCCM/PSBI services, including 1) perceived low quality of service and community trust; 2) HEWs workload and shortage of HEWs; and 3) shortage of supplies.

Low perceived quality of and trust in health post care: HEWs who participated in the IDIs reported that communities' perceived low quality of and trust in health post care delayed care-seeking at facilities as they opted for traditional healers. They also bypassed health posts to seek care at health centers or hospitals. Respondents said:

“They [communities] usually do not believe they will get good service at health posts.” (HEW respondent)

“The community members usually have limited trust in the services at health posts.” (HEW respondent)

Poor health posts infrastructure also affects the healthcare-seeking behaviors of the community:

“… if you have seen the condition of the health posts you visited today, I am sure you have noticed what the roofs look like other health posts and have neither a toilet nor a fence. The rooms are full of dust. They are in bad condition, and the situations the health posts are in will make every client feel disappointed.” (iCCM focal person respondent)

Shortage of drugs: One of the remaining challenges to delivering iCCM/PSBI services was a shortage of supplies and essential drugs. Amoxicillin dispersible tablet was a persistent challenge:

“… the first challenge is related to the shortage of supplies and medicines. Especially the shortage of drugs has been a serious challenge for us to provide the iCCM service.” (iCCM focal person respondent)

Shortage of HEWs, workload, and other competing priorities: Shortage of HEWs and workload were also reported as significant challenges for delivering iCCM/PSBI services. For example, the health posts would be closed when HEWs conduct community and outreach-based services. One HEW respondent said:

“For instance, in this health post, as you can see, I am alone, and when there are campaigns like for the trachoma treatment, I stay on the field for ten consecutive days. So, the health post will

be closed during campaigns and other activities. Closure of health posts affects the health service utilization of community members as they become upset when they get the health post closed.”

Other competing priorities of the government also affected the delivery of iCCM/PSBI services:

“Well, the government is over-engaged with emerging tasks, and when such activities arise, we interrupt iCCM activities and move to the emergent task. So, we would rather focus on these [emergent] tasks and then have our iCCM performance evaluated.” (HEW respondent)

it would be useful to show in a tabe format challenges identified in baseline, imolentation strategies / interventions adopted, which strategies wrkrd the most to improve effective coverage. at individaul, Hs and community laevel

Discussion

The adaptive implementation strategies designed and implemented to maintain quality community-based PSBI treatment in this implementation research effectively engaged community volunteers, HEWs, and the primary health care system, and improved access to and quality delivery of PSBI treatment during the COVID-19 pandemic. The trends in the effective coverage for the appropriate management of PSBI have also significantly improved following the intervention.

The prevalence of PSBI significantly decreased over the implementation period, which is lower than in previous studies in Ethiopia and systematic review and meta-analysis studies [33, 36]. This might be attributed to the implementation strategies that promoted facility birth and essential newborn care practice, as well as community education regarding COVID-19 preventive measures.

This implementation research showed that mothers’ care-seeking behavior for medical care for their sick newborns, as well as intervention-adjusted coverage of receiving appropriate

Clearly state the strategies and interventions hat helped to remove key barriers and why some barrie continued to pursu such that coverage of newborr who took approprite antibiotics was still ;low.

antibiotics, increased significantly. While most mothers sought medical care for their severely sick newborns after the intervention, the coverage of newborns who took appropriate antibiotics is still low, as was also documented in earlier implementation studies [29]. The possible reason for the dropout across the treatment cascade could be the low competence of caregivers.

Designing strategies to improve the quality of care is a critical component of ending preventable newborn mortality and global health leaders explicitly call for the improvement in quality of healthcare services as an integral component to achieve universal health coverage of SDG targets [19]. Evidence shows low levels of treatment-seeking and community misperceptions about the severity of newborn illnesses or quality of care [14, 15], caregivers' lack of recognition of signs of newborn infection and low care-seeking prevalence [37, 38], and poor adherence to iCCM treatment guidelines [39, 40] contributed to low service quality. Strengthening the health system, improved supervision and additional training for providers, and interventions to change the workplace culture have been tested to improve adherence to protocols and quality of health care in developing countries [41]. In addition, for higher effective coverage of PSBI treatment when referral is not feasible, social mobilization strategies targeting families and communities and client-provider interactions need to be strengthened to adhere to treatment protocols and proper counseling and adherence monitoring.

Previous implementation research in Ethiopia demonstrated a higher treatment coverage for PSBI through training health care workers, in-field technical support, performance reviews [25], community engagement [12, 25], strengthening supply chain management [25], standardization of treatment protocols at the health center and health post levels [25], policy dialogue, presence

of state-level sensitization workshop, and establishment of a technical support unit [12, 25]. The qualitative component of this IR affirmed that implementation strategies, particularly the social mobilization interventions, improved communities' awareness about the availability of PSBI services in their vicinity health posts. In this IR, HEWs identified and managed about 26% of PSBI cases at the end-line, which is a significant improvement from 4% at baseline where most families sought care directly from health centers or hospitals, or sought no care at all. Access to PSBI treatment near their homes would help avert difficulties of access to care to facilities in rural areas due to terrain and long distances.

Important strategies to address access and continuum of care challenges include: identification of all pregnancies and antenatal counseling visits, which promote antepartum care and birth preparedness and notification; notification of all deliveries within the first day; and early postnatal home visits to assess newborns, counsel mothers (including danger sign recognition), and refer and/or manage suspected newborn infections for identifying cases and beginning full-course of appropriate antibiotics treatment [42]. In this implementation research, HEWs made about 35 home visits per health post per month and identified an average of three infants per health post per month between April 2021 to May 2022. However, while postpartum home visitation showed improvement, it remains weak in the country. Further strengthening this link will be key for the successful implementation of PSBI case management at a community level across Ethiopia.

In Ethiopia, the iCCM/PSBI treatment program is integrated with the continuum of maternal and child health care framework from pregnancy to childhood and from household to health facilities.

We have learned that integration of strategies into the woreda health system, continuous support systems, and technical oversight were critical for frontline health workers to deliver PSBI services and to create a resilient community health system to provide quality PSBI care during the pandemic. Implementation facilitation and support to the HEP could improve the delivery of PSBI management in the community when hospital referral is not feasible. As the HEP is the intermediary unit between the community and primary care facilities in a woreda health system, strong relationships with stakeholders in the community and health system are also critical for high performance. Strong technical oversight and accountability mechanisms are found to facilitate community trust, communication, and relationships, which influence HEP performance [43]. Previous studies also demonstrated that more frequent and supportive guidance and administrative support, audits with feedback, and checklist-based supervision create an enabling work environment [44, 45] and improve the performance and motivation of primary healthcare workers [46].

Despite observed improvements, remaining barriers impeded optimal iCCM/PSBI service operations, including perceived low quality of service and community trust, low frequency of home visitation, high workloads and shortage of HEWs, and lack of supplies. Most of these challenges could be resolved by woreda and primary healthcare technical support and program oversight. Accordingly, the introduction of continuous audit and feedback systems on the quality of case management, continuous supply of essential drugs, and strategies to alleviate HEWs' workload to deliver quality services should be prioritized.

Measuring trends in coverage for the appropriate management of PSBI by place of treatment (health facility or community) and type of provider are well recognized to assess the quality of the iCCM program to address critical bottlenecks, which limit capacity to provide effective care, including access to and use of care. Although effective coverage has been increasingly used in the evaluation of maternal and child health programs, population-level data on the place of treatment and the type of health provider are scarce in LMICs. To our knowledge, this is the first study that estimates both crude and effective coverage of PSBI management. Another strength of the study is the participatory design and implementation with active engagement of stakeholders that foster ownership and integration of strategies. However, we could not assess the full spectrum of treatment cascade across the treatment continuum, nor estimate input-adjusted or quality-adjusted coverage. As such, linking data from households and health facilities could produce meaningful estimates of coverage that could help to access the input and quality-adjusted coverages [34].

Conclusions

The COVID-19 adaptive implementation strategies included training and coaching, creation of support systems and technical support units, supply strengthening of essential commodities, and community awareness creation about PSBI and COVID-19; these strategies led to improved care-seeking behavior of mothers for their sick young infants and improved the quality of PSBI management. Support systems were critical for frontline health workers to deliver PSBI services and create a resilient community health system to provide quality PSBI care during the pandemic. Continuous audit and feedback systems on the quality of case management, continuous supply

of essential drugs, and strategies to alleviate HEWs’ workload to deliver quality services should be prioritized. In addition, social mobilization strategies targeting families and communities, as well as client-provider interactions need to be strengthened to further improve effective coverage of community-based management of PSBI when referral is not feasible.

List of abbreviations

BMGF	Bill & Melinda Gates Foundation
CFIR	Consolidated Framework for Implementation Research
CHIS	Community Health Information System
COVID-19	Coronavirus
EPOC	Effective Practice and Organization of Care
ERIC	Expert Recommendations for Implementation Change
HEP	Health Extension Program
HEW	Health Extension Worker
iCCM	integrated community case management of childhood illnesses and newborn care
IDI	in-depth interview
IRLM	Implementation Research Logic Model
JSI	JSI Research & Training Institute Inc.
KOICA	Korea International Cooperation Agency
L10K	Last Ten Kilometers Project
LMIC	low-and-middle-income countries
NMR	neonatal mortality rate
PSBI	possible serious bacterial infection
RE-AIM	Reach, Effectiveness, Adoption, Implementation, and Maintenance
SDG	sustainable development goal
USAID	United States Agency for International Development
WDA	Women Development Army
WHO	World Health Organization

Declarations

Consent for publication

Not applicable

Availability of data and materials

The dataset used and analyzed during this study is included as supplementary information to this article (Additional file 3).

Competing interests

The authors declare that they have no competing interests. The authors [GT, NF, DE, and WB] work for JSI Research & Training Institute, Inc., a commercial company. We declare that this commercial affiliation does not alter our adherence to the journal's policies on sharing data and materials. One of the authors of this manuscript (HM) works for the Gates Foundation. We would like to declare that we do not have any conflict of interest with the Gates Foundation-paid staff in preparing this manuscript.

Funding

The article write-up and publication fee were supported by the Bill & Melinda Gates Foundation, Investment Number INV-024241. JSI Research & Training Institute, Inc. has helped us in the form of salaries for authors [GT, NF, DE, and WB]. However, none of the funders played a role in the study design, data collection, analysis, decision to publish, or manuscript preparation.

Authors' contributions

GT, NF, DE, WB, LRH, and HM: conceived the study design and methods. GT, NF, DE, WB, TG, HM, and LH: formulated the research question, analysis method, interpretation, and critical review. GT, NE, and LH: conceptualized the paper. GT: drafted the manuscript. All authors synthesized, interpreted, and provided critical review. All authors read and approved the final manuscript.

Acknowledgments

We thank the Bill & Melinda Gates Foundation for funding this implementation research. The implementation of this survey would not have been possible without the support of Ethiopia's Ministry of Health, Amhara and Oromia Regional Health Bureaus, National Child Health Technical

Working Group, National reproductive, maternal, newborn, child, adolescent health and nutrition Research Advisory Council Child Health and Immunization group, and other development partners (Transform Primary Health Care, Fenot project, UNICEF, KOICA, and USAID). We acknowledge the interviewers and the supervisors for their hard work, dedication, and for accomplishing the fieldwork on schedule. Finally, we take this opportunity to extend our gratitude to all study participants who took their time to respond to the survey questionnaires and provide us with invaluable information. Alexandra Kamberos is well-acknowledged for editing the manuscript.

References

1. United Nations Inter-agency Group for Child Mortality Estimation (UN IGME). Levels & Trends in Child Mortality: Report 2022, Estimates developed by the United Nations Inter-agency Group for Child Mortality Estimation. New York: United Nations Children's Fund; 2023.
2. Ethiopian Public Health Institute (EPHI) [Ethiopia], ICF. Ethiopia Mini Demographic and Health Survey 2019: Final Report. Rockville, Maryland, USA: EPHI and ICF; 2021.
3. Perin J, Mulick A, Yeung D, Villavicencio F, Lopez G, Strong KL, et al. Global, regional, and national causes of under-5 mortality in 2000–19: an updated systematic analysis with implications for the Sustainable Development Goals. The Lancet Child & Adolescent Health. 2022;6(2):106-15. doi: https://doi.org/10.1016/S2352-4642(21)00311-4.
4. Wammanda RD, Adamu SA, Joshua HD, Nisar YB, Qazi SA, Aboubaker S, et al. Implementation of the WHO guideline on treatment of young infants with signs of possible serious bacterial infection when hospital referral is not feasible in rural Zaria, Nigeria: Challenges and solutions. PloS one. 2020;15(3):e0228718.
5. Abdella YM, Bekele A, Mathewos B, Tadesse Y, Wall S, Gardner H, et al. Do Caretakers of Sick Young Infants with Possible Serious Bacterial Infection Adhere to Referrals from Health Posts to Health Centers? Ethiopian Medical Journal. 2019(3).
6. WHO. Guideline: Managing possible serious bacterial infection in young infants when referral is not feasible. Switzerland: World Health Organization; 2015.
7. MOH. National Strategy for Newborn and Child Survival in Ethiopia: 2016-2020. Addis Ababa, Ethiopia: Ministry of Health, Ethiopia; 2015.
8. Estifanos AS, Kazmi K, Morris SK. Could COVID-19 Reverse the Modest Gains Made in Newborn Health in Ethiopia? Maternal and Child Health Journal. 2021;25(6):849-54.
9. Gebreegziabher SB, Marrye SS, Kumssa TH, Merga KH, Feleke AK, Dare DJ, et al. Assessment of maternal and child health care services performance in the context of COVID-19 pandemic in Addis Ababa, Ethiopia: evidence from routine service data. Reproductive Health. 2022;19(1):42. doi: 10.1186/s12978-022-01353-6.
10. Tareke KG, Lemu YK, Feyissa GT. Exploration of facilitators of and barriers to the community-based service utilization for newborn possible serious bacterial infection management in Debre Libanos District, Ethiopia: descriptive qualitative study. BMC pediatrics. 2020;20(1):1-14.
11. Gebretsadik A, Melaku N, Haji Y. Community Acceptance and Utilization of Maternal and Community-Based Neonatal Care Services Provided by Health Extension Workers in Rural Sidama Zone: Barriers and Enablers: A Qualitative Study. Pediatric health, medicine and therapeutics. 2020;11:203-17. doi: 10.2147/phmt.S254409.
12. Leul A, Hailu T, Abraham L, Bayray A, Terefe W, Godefay H, et al. Innovative approach for potential scale-up to jump-start simplified management of sick young infants with possible serious bacterial infection when a referral is not feasible: Findings from implementation research. PloS one. 2021;16(2):e0244192.
13. Avan BI, Berhanu D, Mekonnen Y, Beaumont E, Tomlin K, Allen E, et al. Embedding Community-Based Newborn Care in the Ethiopian health system: lessons from a 4-year programme evaluation. Health policy and planning. 2021;36(Supplement_1):i22-i32. doi: 10.1093/heapol/czab085.

14. Tareke KG, Lemu YK, Yidenekal SA, Feyissa GT. Community's perception, experiences and health seeking behavior towards newborn illnesses in Debre Libanos District, North Shoa, Oromia, Ethiopia: Qualitative study. PLoS One. 2020;15(1):e0227542. doi: 10.1371/journal.pone.0227542.
15. Asfaha MD, Comeau DL, Spangler SA, Spratt BL, Alamineh L, Gobezayehu AG, et al. Neonatal care and community-level treatment seeking for possible severe bacterial infection (PSBI) in Amhara, Ethiopia. BMC health services research. 2020;20:1-12.
16. Tiruneh GT, Nigatu TG, Magge H, Hirschhorn LR. Using the Implementation Research Logic Model to design and implement community-based management of possible serious bacterial infection during COVID-19 pandemic in Ethiopia. BMC Health Services Research. 2022;22(1):1515. doi: 10.1186/s12913-022-08945-9.
17. Yakob B, Gage A, Nigatu TG, Hurlburt S, Hagos S, Dinsa G, et al. Low effective coverage of family planning and antenatal care services in Ethiopia. International Journal for Quality in Health Care. 2019;31(10):725-32. doi: 10.1093/intqhc/mzy251.
18. UN: Sustainable Development Goals. 17 goals to transform our world. (2015). Accessed.
19. UNDP. Sustainable development goals. Geneva, Switzerland: United Nations; 2015.
20. MOH. Ethiopia Health Sector Transformation Plan (2015/16-2019/2020). Addis Ababa, Ethiopia: Ministry of health, Ethiopia; 2015.
21. Glasgow RE, Vogt TM, Boles SM. Evaluating the public health impact of health promotion interventions: the RE-AIM framework. American journal of public health. 1999;89(9):1322-7.
22. Damschroder LJ, Aron DC, Keith RE, Kirsh SR, Alexander JA, Lowery JC. Fostering implementation of health services research findings into practice: a consolidated framework for advancing implementation science. Implementation Science. 2009;4(1):50. doi: 10.1186/1748-5908-4-50.
23. Taxomony E. Effective Practice and Organisation of Care (EPOC). 2015.
24. Proctor EK, Powell BJ, McMillen JC. Implementation strategies: recommendations for specifying and reporting. Implementation Science. 2013;8(1):1-11.
25. Berhane M, Girma T, Tesfaye W, Jibat N, Abera M, Abrahim S, et al. Implementation research on management of sick young infants with possible serious bacterial infection when referral is not possible in Jimma Zone, Ethiopia: Challenges and solutions. PLOS ONE. 2021;16(8):e0255210. doi: 10.1371/journal.pone.0255210.
26. Goyal N, Rongsen-Chandola T, Sood M, Sinha B, Kumar A, Qazi SA, et al. Management of possible serious bacterial infection in young infants closer to home when referral is not feasible: Lessons from implementation research in Himachal Pradesh, India. PLOS ONE. 2020;15(12):e0243724. doi: 10.1371/journal.pone.0243724.
27. Awasthi S, Kesarwani N, Verma RK, Agarwal GG, Tewari LS, Mishra RK, et al. Identification and management of young infants with possible serious bacterial infection where referral was not feasible in rural Lucknow district of Uttar Pradesh, India: An implementation research. PloS one. 2020;15(6):e0234212.
28. Roy S, Patil R, Apte A, Thibe K, Dhongade A, Pawar B, et al. Feasibility of implementation of simplified management of young infants with possible serious bacterial infection when referral is not feasible in tribal areas of Pune district, Maharashtra, India. PloS one. 2020;15(8):e0236355.

Please also add
Mukhopadhyay R, Arora NK, Sharma PK, Dalpath S, Limbu P, Kataria G, et al. (2021) Lessons from implementation research on community management of Possible Serious Bacterial Infection (PSBI) in young infants (0-59 days), when the referral is not feasible in Palwal district of Haryana, India. PLoS ONE 16(7): e0252700. https://doi.org/10.1371/journal. pone.0252700

29. Guenther T, Mopiwa G, Nsona H, Qazi S, Makuluni R, Fundani CB, et al. Feasibility of implementing the World Health Organization case management guideline for possible serious bacterial infection among young infants in Ntcheu district, Malawi. PloS one. 2020;15(4):e0229248.
30. Ariff S, Soofi SB, Suhag Z, Chanar S, Bhura M, Ahmed I, et al. Innovation to Increase Treatment Coverage of Possible Serious Bacterial Infections in Young Infants When a Referral is Not Feasible Implemented in the Existing Health System in Sindh, Pakistan. Preprint. 2021.
31. Ayede AI, Ashubu OO, Fowobaje KR, Aboubaker S, Nisar YB, Qazi SA, et al. Management of possible serious bacterial infection in young infants where referral is not possible in the context of existing health system structure in Ibadan, south-west Nigeria. PloS one. 2021;16(3):e0248720.
32. Rahman AE, Herrera S, Rubayet S, Banik G, Hasan R, Ahsan Z, et al. Managing possible serious bacterial infection of young infants where referral is not possible: Lessons from the early implementation experience in Kushtia District learning laboratory, Bangladesh. PLOS ONE. 2020;15(5):e0232675. doi: 10.1371/journal.pone.0232675.
33. Berhanu D, Okwaraji YB, Defar A, Bekele A, Lemango ET, Medhanyie AA, et al. Does a complex intervention targeting communities, health facilities and district health managers increase the utilisation of community-based child health services? A before and after study in intervention and comparison areas of Ethiopia. BMJ open. 2020;10(9):e040868.
34. Riese S, Shireen Assaf, Thomas Pullum. Measurement Approaches for Effective Coverage Estimation. DHS Methodological Reports No. 31. Rockville, Maryland, USA: ICF; 2021.
35. Marsh AD, Muzigaba M, Diaz T, Requejo J, Jackson D, Chou D, et al. Effective coverage measurement in maternal, newborn, child, and adolescent health and nutrition: progress, future prospects, and implications for quality health systems. The Lancet Global Health. 2020;8(5):e730-e6. doi: 10.1016/S2214-109X(20)30104-2.
36. Seale AC, Blencowe H, Manu AA, Nair H, Bahl R, Qazi SA, et al. Estimates of possible severe bacterial infection in neonates in sub-Saharan Africa, south Asia, and Latin America for 2012: a systematic review and meta-analysis. The Lancet infectious diseases. 2014;14(8):731-41.
37. Berhane M, Yimam H, Jibat N, Zewdu M. Parents' knowledge of danger signs and health seeking behavior in newborn and young infant illness in Tiro Afeta district, Southwest Ethiopia: a community-based study. Ethiopian Journal of health sciences. 2018;28(4).
38. Getachew T, Assebe Yadeta T, Gereziher T, Eyeberu A, Dheresa M. Determinants of maternal knowledge on neonatal danger signs and care-seeking practices in a rural area of southeastern Ethiopia. International Health. 2021;14(6):610-8. doi: 10.1093/inthealth/ihab084.
39. Daka DW, Wordofa MA, Woldie M, Persson LÅ, Berhanu D. Quality of clinical assessment and management of sick children by Health Extension Workers in four regions of Ethiopia: A cross-sectional survey. PLOS ONE. 2020;15(9):e0239361. doi: 10.1371/journal.pone.0239361.
40. Getachew T, Abebe SM, Yitayal M, Persson LÅ, Berhanu D. Assessing the quality of care in sick child services at health facilities in Ethiopia. BMC health services research. 2020;20:1-12.
41. Leonard KL, Masatu MC. Changing health care provider performance through measurement. Social Science & Medicine. 2017;181:54-65. doi: https://doi.org/10.1016/j.socscimed.2017.03.041.

42. MOH. National Implementation Guideline For Integrated Community Case Management of Childhood Illnesses and Newborn Care. Addis Ababa, Ethiopia: Ministry of Health, Ethiopia; 2017.
43. Kok MC, Kea AZ, Datiko DG, Broerse JEW, Dieleman M, Taegtmeyer M, et al. A qualitative assessment of health extension workers' relationships with the community and health sector in Ethiopia: opportunities for enhancing maternal health performance. Human resources for health. 2015;13(1):80. doi: 10.1186/s12960-015-0077-4.
44. Jaskiewicz W, Tulenko K. Increasing community health worker productivity and effectiveness: a review of the influence of the work environment. Human Resources for Health. 2012;10(1):38. doi: 10.1186/1478-4491-10-38.
45. Tiruneh GT, Karim AM, Yihun BY, Birhanu BG, Amaha A, Mathiwos B, et al. Effectiveness of Supervision on the Consistency of Neonatal Sepsis Management Skills of Health Extension Workers. Ethiopian Medical Journal. 2019.
46. Rabbani F, Shipton L, Aftab W, Sangrasi K, Perveen S, Zahidie A. Inspiring health worker motivation with supportive supervision: a survey of lady health supervisor motivating factors in rural Pakistan. BMC health services research. 2016;16(1):397.

Supplementary information

Additional file 1: Survey questionnaire.xlsx. A household survey questionnaire we used to collect information from study participants. The first sheet contains variable definitions (data dictionary) in English and local languages, and the second sheet contains variable answer choices.

Additional file 2: In-depth interview guides.docx. These are interview guides for service providers

Additional file 3: Survey dataset.xls. This is household survey data with variables and the values we used for this analysis.

Click here to access/download;Figure;Figure 2.tif

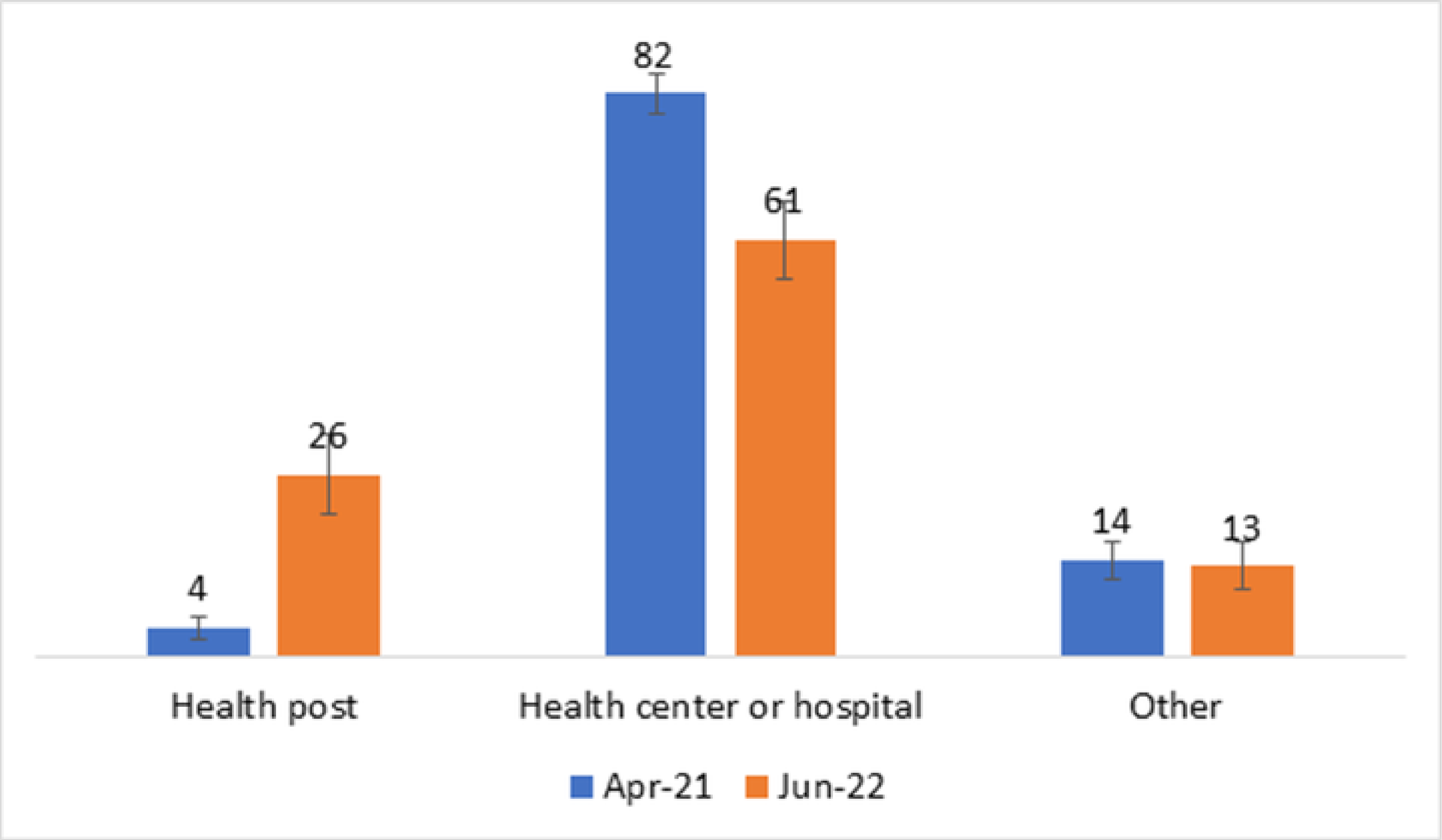

Click here to access/download;Figure;Figure 3.tif

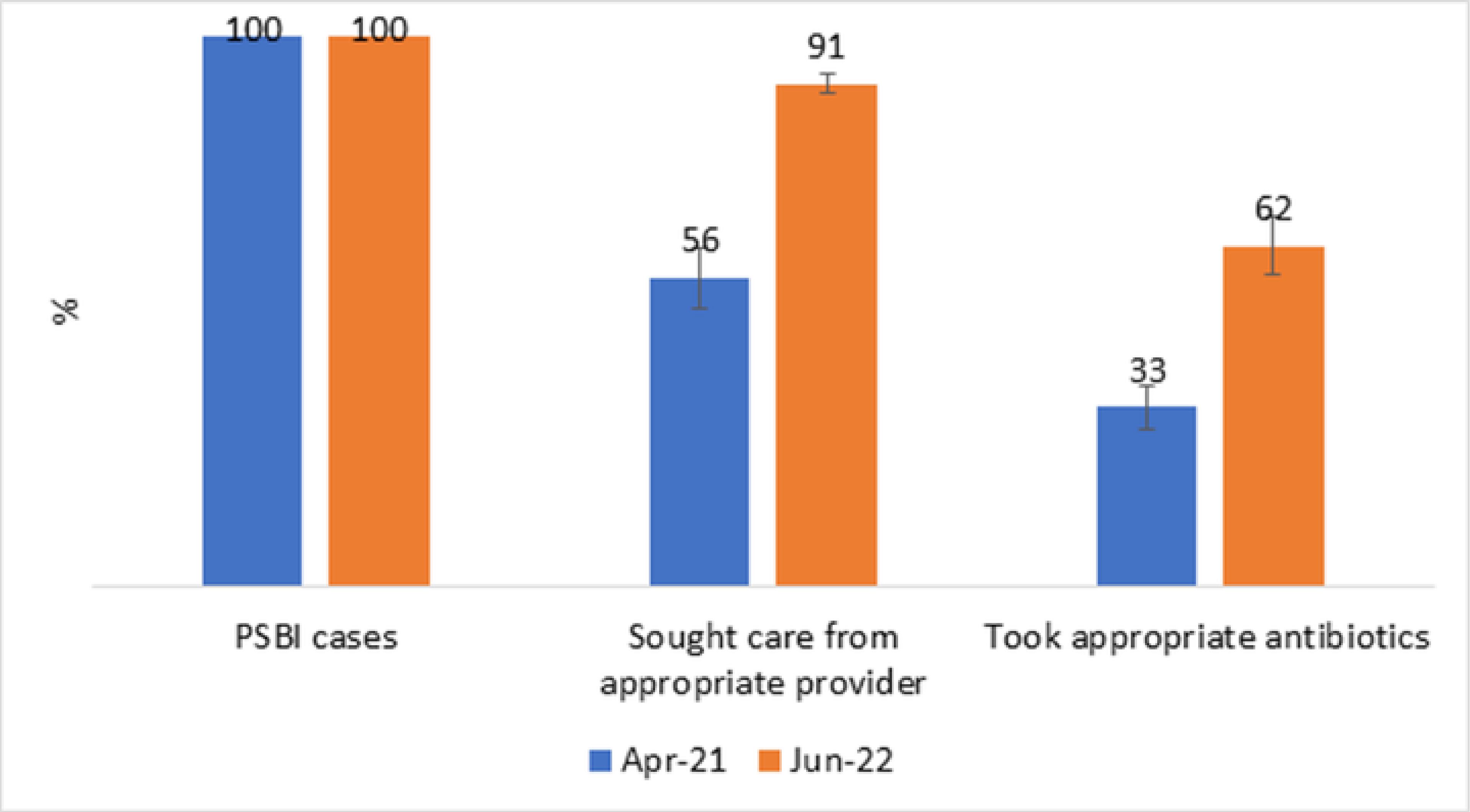

Click here to access/download;Figure;Figure 1.tif

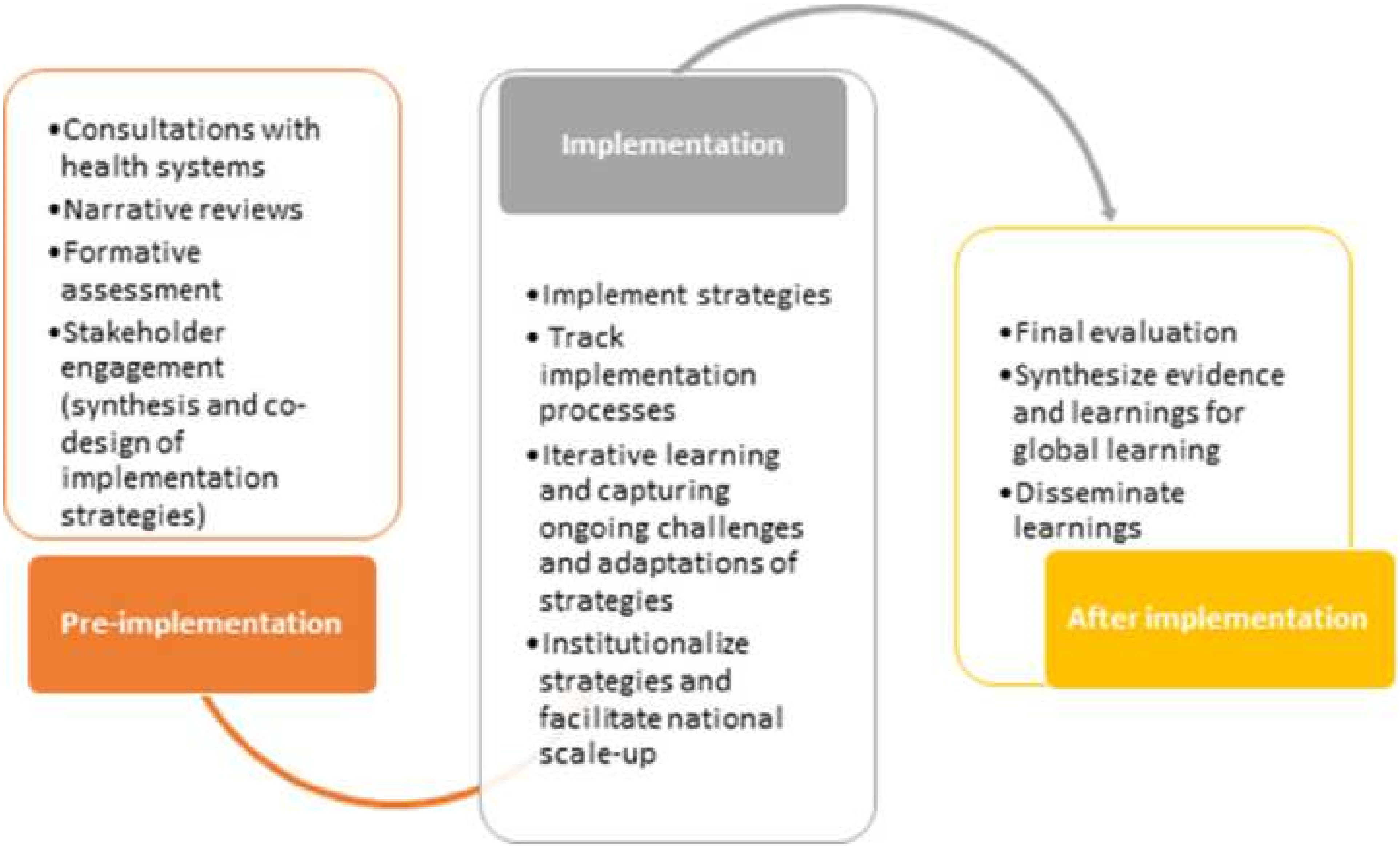

Click here to access/download;Figure;Additional file 2.docx

In-depth interview guides for service providers (Health center staff and HEWs)
End line evaluation of the PSBI implementation research (IR), qualitative component

I	Identification	
1	Questionnaire ID	
2	Area Identification	
3	Name of Woreda/Zone/Region	
4	Name of facility	
5	Name of moderator	
6	Name of a note taker	
7	Date of discussion	
8	Participant #	
9	Audio File #	
10	Start time:	______:________
11	End time:	____:______
12	Transcriber	

SN	Questions	Probe
	**Fidelity**	
1	Can you please describe the demand generation/SBCC activities you are doing on iCCM/ newborn health? What are the strategies used to raise awareness? If none, please explain why.<br><br>How is the engagement of communities/WDA networks in the implementation of integrated community-based case management (iCCM) of common childhood illnesses/possible serious bacterial infection (PSBI)?	What are some of the biggest challenges about SBCC activities for newborn care?
2	How do you describe the strength of the support you got from the PHC and the project as well as your efforts to address barriers to deliver iCCM/PSBI during COVID-19?	What was changed?<br><br>Was the support you got from the project and PHC helpful?<br><br>What could have been done differently?
3	Did do you get the support system helpful (mentorship and on-site coaching methods, supervision, technical support, PHCU level PRCMM, etc.) to enhance your skills [that is HEWs' skills] over the traditional off-site training and woreda level review meetings?	**Probe** for acceptability of the strategies (**for HEWs only)**

		How could the support system be improved?
4	How eCHIS implementation helps you with iCCM service delivery?	**Probe for advantages:** case identification? quality of iCCM case management? retrieval of client records? data quality? client appointment scheduling and/or defaulter tracing?<br><br>**Ask why?**<br><br>**Probe for areas of strengthening?**
5	How was the introduction of the iCCM/CBNC module into eCHIS affected the iCCM/CBNC service delivery?<br><br>**For Lume woreda only**	**Probe for advantages:** identification and registration of sick newborns; clinical and referral decision support; follow-up of sick child treatment and/or defaulter tracing; retrieval of client records; appointment scheduling; client satisfaction<br><br>**Ask why?**<br><br>**Probe for areas of strengthening?**
	**Implementation challenges**	
6	Describe the main issues faced by the health system to identify and treat neonatal infections in the community. What are the critical factors affecting the **delivery of iCCM** to clients?	**Probe**; lack of competence of HEWs, shortage of supplies and commodities; weak support system; low community demand?<br><br>**Regional/national state of emergency and conflict in the northern part of the country?**<br><br>How has that changed during COVID?

7	How do you think the COVID-19 pandemic and/or COVID-19 response measures affect the uptake and/or delivery of iCCM services?	**Probe:** Has COVID-19 affected your daily routines; your work on newborn; the community in terms of livelihood and vulnerability for newborn care-seeking<br><br>How has that changed over time
	**Adoption and reach/effectiveness**	
8	Explain to us any changes you observed as the result of the implementation of the COVID-19 adaptive iCCM implementation strategies? – What impact has the strategies had? What are the particular features of the strategies that made a difference?	**Probe** for support system and linkages, motivation and competence of HEWs, community engagement and awareness creation?<br><br>What do you think are the reasons for non-significant changes?
	**Maintenance and sustainability**	
9	What are the high-level benefits that are attributable to this support/IR?	
10	Please explain to us the feasibility of this support/ IR for national scale-up?	What features could easily be integrated into the existing system? Which not?
11	How are the activities/efforts embedded in the PHC and woreda routines/work streams?What implementation strategies are incorporated with the PHC and woreda annual work plan?	
12	Do you have anything think is important to tell us that we have not asked you?	

Thanks!!

Click here to access/download
Supporting Information
Additional file 3.csv

Click here to access/download
Supporting Information
Additional file 1.xlsx

Click here to access/download
Supporting Information
StaRI-checklist-for-author-completion.docx

Click here to access/download
Supporting Information
STROBE_checklist_cross-sectional.docx

Click here to access/download
Supporting Information
COREQ_Checklist_qualitative.DOCX
