# Supplementary material for: Effect of community-based newborn care implementation strategies on access to and effective coverage of possible serious bacterial infection (PSBI) treatment for sick young infants during COVID-19 pandemic
Source: PLoS One. 2024 Mar 25;19(3):e0300880. doi: 10.1371/journal.pone.0300880 (PMC10962833; doi:10.1371/journal.pone.0300880)
Supplement: Attachment — Submitted filename: Point-by-point response.docx [file pone.0300880.s005.docx]

**Cover letter**

**PONE-D-23-12889**

**Effect of community-based newborn care implementation strategies on access to and effective coverage of possible serious bacterial infection (PSBI) treatment for sick young infants during COVID-19 pandemic**

Dear editors,

*We, the authors, would like to thank the reviewers and the editor for their valuable comments. Our point-by-point responses to the reviewers and editor are below each of the comments in italics. We also reviewed to ensure that this version of the manuscript conforms to the journal style.*

**Journal Requirements:**

When submitting your revision, we need you to address these additional requirements.

1. Please ensure that your manuscript meets PLOS ONE's style requirements, including those for file naming. The PLOS ONE style templates can be found at

<https://journals.plos.org/plosone/s/file?id=wjVg/PLOSOne_formatting_sample_main_body.pdf> and

<https://journals.plos.org/plosone/s/file?id=ba62/PLOSOne_formatting_sample_title_authors_affiliations.pdf>

*Response: Thank you for the comments. The manuscript is formatted according to the journal style requirements.*

2. We noticed you have some minor occurrence of overlapping text with the following previous publication(s), which needs to be addressed:

<https://bmchealthservres.biomedcentral.com/articles/10.1186/s12913-022-08945-9>

<https://publications.jsi.com/JSIInternet/Inc/Common/_download_pub.cfm?id=24907&lid=3>

In your revision ensure you cite all your sources (including your own works), and quote or rephrase any duplicated text outside the methods section. Further consideration is dependent on these concerns being addressed.

*Response: Our apologies and thank you for alerting us. In this version, we revised it accordingly and have checked to ensure that any repeated language is marked as quote and referenced.*

3. Thank you for stating the following in the Acknowledgments Section of your manuscript:

"We thank the Bill & Melinda Gates Foundation for funding this implementation research."

We note that you have provided additional information within the Acknowledgements Section that is not currently declared in your Funding Statement. Please note that funding information should not appear in the Acknowledgments section or other areas of your manuscript. We will only publish funding information present in the Funding Statement section of the online submission form.

Please remove any funding-related text from the manuscript and let us know how you would like to update your Funding Statement. Currently, your Funding Statement reads as follows:

"The article write-up and publication fee were supported by the Bill & Melinda Gates Foundation, Investment Number INV-024241. JSI Research & Training Institute, Inc. has helped us in the form of salaries for authors [GT, NF, DE, and WB]. However, none of the funders played a role in the study design, data collection, analysis, decision to publish, or manuscript preparation."

Please include your amended statements within your cover letter; we will change the online submission form on your behalf.

*Response: Thank you for the comments. The funding information is removed from the manuscript and the Acknowledgment section. The Funding Statement submitted in the online submission form is correct.*

4. Thank you for stating the following in the Competing Interests section:

"The authors declare that they have no competing interests. The authors [GT, NF, DE, and WB] work for JSI Research & Training Institute, Inc., a commercial company. We declare that this commercial affiliation does not alter our adherence to the journal's policies on sharing data and materials. One of the authors of this manuscript (HM) works for the Gates Foundation. We would like to declare that we do not have any conflict of interest with the Gates Foundation-paid staff in preparing this manuscript."

Please confirm that this does not alter your adherence to all PLOS ONE policies on sharing data and materials, by including the following statement: "This does not alter our adherence to  PLOS ONE policies on sharing data and materials.” (as detailed online in our guide for authors <http://journals.plos.org/plosone/s/competing-interests>). If there are restrictions on sharing of data and/or materials, please state these. Please note that we cannot proceed with consideration of your article until this information has been declared.

Please include your updated Competing Interests statement in your cover letter; we will change the online submission form on your behalf.

*Response: Thank you for the comments. It is now updated as follows;*

*"The authors declare that they have no competing interests. The authors [GT, NF, DE, and WB] work for JSI Research & Training Institute, Inc., a commercial company. This does not alter our adherence to PLOS ONE policies on sharing data and materials. One of the authors of this manuscript (HM) works for the Gates Foundation. We would like to declare that we do not have any conflict of interest with the Gates Foundation-paid staff in preparing this manuscript."*

5. Please upload a new copy of Figure 1 as the detail is not clear. Please follow the link for more information: <https://blogs.plos.org/plos/2019/06/looking-good-tips-for-creating-your-plos-figures-graphics/>" <https://blogs.plos.org/plos/2019/06/looking-good-tips-for-creating-your-plos-figures-graphics>

*Response: Thank you for the comments. The revised version is uploaded.*

6. Please review your reference list to ensure that it is complete and correct. If you have cited papers that have been retracted, please include the rationale for doing so in the manuscript text, or remove these references and replace them with relevant current references. Any changes to the reference list should be mentioned in the rebuttal letter that accompanies your revised manuscript. If you need to cite a retracted article, indicate the article’s retracted status in the References list and also include a citation and full reference for the retraction notice.

*Response: Thank you for the comments. The references are reviewed.*

Reviewers' comments:

**Review Comments to the Author**

Reviewer #1: Methods section:
1. Around line 139, it would have been good if they clearly describe the roles and responsibilities of each in detail so that readers understand the different categories of role players (like the HEWs did this and that, implementers did this and that, etc.). Plus, the intervention package should have also been elaborated so that one understands what has been done differently that led to the changes in the outcome variables.

*Response: Thank you for the valid comments. We presented the strategies, details of the activities, and roles of stakeholders in tabular form (see additional Table 1, page 8)*

2. Line 147: Why did you use prevalence estimate of 5%? There are different figures as to the prevalence (estimation) of PSBI in the first 2months of life.

*Response:* We agree that *different studies have reported different values of prevalence of PSBI that range from 5-6% at baseline and 11-13% at end line (Berhanu et al., 2020).* A *systematic review and meta-analysis that included data from 22 studies reported PSBI incidence risk estimate of 6% in the sub-Saharan Africa region and overall pooled estimate of 7.6% (Seale et al., 2014). Some studies reported more than 10% prevalence which could be due to the inclusion of local infection signs (Puri et al., 2021). We took 5% prevalence based on the baseline estimate of a large household study in Ethiopia (Berhanu et al., 2020) as our primary objective was to see the effect of the IR on improved care-seeking for PSBI treatment Our sample size was determined based on the prevalence of care-seeking for PSBI, 56% not 5% of PSBI. We used the 5% prevalence estimate to check whether we could get an adequate sample of mothers seeking care for PSBI in the study areas.*

3. Outcome measures: line 182: receipt of appropriate antibiotics: What does this mean? Just a dose, 2 doses, 5 doses or what? Plus, would you call appropriate/effective if an infant receives amoxicillin or gentamicin alone? What about those who received other antibiotics like ampicillin?

*Response: In this study, appropriate treatment for PSBI is defined as the recipient of antibiotics for the treatment of PSBI according to the national iCCM/IMNCI standards of treatment which includes gentamicin injection and amoxicillin dispersible tablet. Accordingly, the intervention-adjusted coverage of PSBI treatment was therefore calculated counting appropriate treatment as recipient of these antibiotics irrespective of the completion of the full course treatment. We also did not count those who took inappropriate treatment or unnecessary antibiotics such as tetracycline eye ointments, antimalarial drugs, ORS, vitamin A supplements, and/ or traditional remedies. While adherence-adjusted would include completion of treatment that is gentamicin injection for 2 days and amoxicillin tablet for 7 days for full course completion of the treatment which is not included in this analysis. The manuscript is revised accordingly to clarify this. See page 11, lines 176-189.*

Results and discussion sections:
4. Line 220: Could the difference in the background characteristics have an effect on the outcome variables? Can you highlight this in the discussion as well?

*Response: Definitely, differences in the background characteristics, or other developmental inputs like road construction that influence MNH could be time-invariant confounders and bias the observed outcome. These are now discussed in the discussion section of the revised version (Page 23, lines 403-409) of the manuscript as presented below.*

*“Time-invariant confounders such as changes in the background characteristics including maternal education, parity, and distance to facility over time or other developmental inputs (road, health facility construction, etc.) that influence maternal and newborn health could bias the observed outcome. However, as this was an implementation research, we were continuously monitoring and adapting and/or modifying strategies to respond to contextual factors. As such, the observed effect could be explained by the implementation intensity of the strategies than the changes in developmental inputs.”*

5. Line 226-232: Instead of the change in the prevalence of PSBI, did you try look at the rate of detection (compared to the estimated/expected) of PSBI before and after the implementation research which could also indicate the changes with regard to the IR?

*Response: Thank you again for the valid comments. We tracked monthly detection rates of PSBI from facility records and monitor the effect of IR on PSBI treatment. In this paper, we presented the changes in the prevalence and care-seeking behavior of mothers for PSBI (from 2 rounds of household surveys).*

6. Changes in the prevalence of PSBI and care seeking behaviour: What specific strategies did you carry out which have led to these change? Promoting institutional deliveries and improving ENCs have already been implemented for long time but with no significant changes and I think it is well known that the intensive COVID 19 prevention strategies did last only for few weeks/months specially in the rural areas. So, attributing these to the factors that led to changes is somehow implausible.

*Response: The support system, technical oversight from the HC and woreda health office were designed to help HEWs and community volunteers to engage into their routine duties. In addition, this approach of mapping strategies to address identified challenges facilitated integration of PSBI treatment into the woreda and PHC work stream (support system, performance review, and planning and budgeting). We have included the following sentences on the manuscript to make it clear.*

*“The qualitative component of the study supported this. The technical oversight and support system from woreda and PHC over community health care systems effectively engaged community volunteers and HEWs into their routine duties leading to improved linkages between communities and health facilities and delivery of PSBI treatment. In addition, facilitating integration of PSBI management into the woreda and PHC work stream (support system, performance review, and planning and budgeting) improved fidelity and sustained implementation. Previous studies also reported that such strategies activated primary health care and community volunteers to treat PSBI (Avan et al., 2021; Berhane et al., 2021; Mukhopadhyay et al., 2021).” Page 19, lines 313-320*

7. Changes in the place of care seeking: Is this really because the care givers wanted to get the service at the HPs or because they are forced to get the service there? Did you try to understand the reason for this from the care givers as well as the HEWs/HCWs perspectives? I think the recommendation for PSBI treatment is still hospitalization and treatment at HP/HC is only if referral is not possible/feasible.

*Response: Both the qualitative and quantitative interviews with mothers revealed it is the communities’ awareness about the availability of PSBI services in their vicinity health posts that improved care seeking at HPs.*

Conclusion/recommendations:
8. These challenges you have figured out have long been identified by the several prior studies as well. What should be done differently to tackle these challenges for the subsequent successful implementation of such strategies?

*Response: Thank you again for the valid comments. Optimizing the integration of PSBI management into the woreda and PHC health system and continuous technical oversight would be helpful. Strong technical oversight and accountability mechanisms were found to foster community trust, effective communication, and positive relationships; all of which contribute to improved health system performance (Kok et al., 2015). To ensure successful implementation and optimal integration of management of PSBI within the health system, several critical factors need to be addressed. These include providing continued coaching and support systems for frontline health workers, ensuring technical oversight from the district/county health teams, and maintaining an adequate supply of essential commodities. The paragraph is edited accordingly, page 22, lines 378-390.*

*“Despite observed improvements, remaining barriers impeded optimal iCCM/PSBI service operations, including perceived low quality of service and community trust, low frequency of home visitation, high workloads and shortage of HEWs, and lack of supplies. Most of these challenges could be resolved by woreda and primary healthcare technical support and program oversight. Strong technical oversight and accountability mechanisms were found to foster community trust, effective communication, and positive relationships; all of which contribute to improved health system performance (Kok et al., 2015). To ensure successful implementation and optimal integration of management of PSBI within the health system, several critical factors need to be addressed. These include providing continued coaching and support systems for frontline health workers, ensuring technical oversight from the district/county health teams, and maintaining an adequate supply of essential commodities. Accordingly, the introduction of continuous audit and feedback systems on the quality of case management, continuous supply of essential drugs, and strategies to alleviate HEWs’ workload to deliver quality services should be prioritized.”*

Reviewer #2: The paper is well written and can be published after some minor comments have been addressed.
1. Clear Explanation of iCCM strategy would be useful specifically with the roles of different facilities and providers - especially the role of HEW vs WDA and HEW vs. Nurse. How is this strategy different from the current implementation strategies applicable in the country.

*Response:* *Thanks a lot for the comments. The following paragraph is added to explain the iCCM strategy and the roles of HEWs and CHWs.*

*“HEWs and WDAs are responsible for educating mothers about early recognition of illness in newborns, and prompt care-seeking assessing danger signs in newborns during postnatal home visits (MOH, 2017), In addition, community-based treatment of PSBI provided by HEWs, integrated with the iCCM program. HEWs are trained to identify newborn infections and provide treatment when referral is not feasible.” Page 6, lines 108-112*

2. While barriers and strategies have been referred to in the other paper by the authors, a better explanation of these in figure 1, would be useful to understand what strategies were implemented and what worked and others did not to increase the effective coverage.

*Response: Thank you for the valid comments. We have presented the strategies, details of the activities, and roles of role players in tabular form (see additional Table 1)*

3. WHile extensive household survey seems to have been done, not much results of the survey can be seen in the paper.

*Response: Thank you again for the comments. The study interviewed women about their sick newborn and about their own health during pregnancy, childbirth, and postpartum period. As the primary objective of the study was averting the impact of COVID-19 and other system bottlenecks on newborn health, in this paper, we presented the strategies designed and implemented, effect of the strategies of PSBI treatment, ongoing challenges for the treatment of PSBI, and changes observed in the uptake of PSBI treatment.*

Some other comments have been added in text boxes in the attached paper.

- Line 191- would be useful to define appropriate treatment. Is it 7 days of antibiotics or partial treatment os also considered appropriate?

*Response: In this study, appropriate treatment for PSBI is defined as the recipient of antibiotics for the treatment of PSBI according to the national iCCM/IMNCI standards of treatment which includes gentamicin injection and amoxicillin dispersible tablet. Accordingly, the intervention-adjusted coverage of PSBI treatment was therefore calculated counting appropriate treatment as recipient of these antibiotics irrespective of the completion of the full course treatment. We also did not count those who took inappropriate treatment or unnecessary antibiotics such as tetracycline eye ointments, antimalarial drugs, ORS, vitamin A supplements, and/ or traditional remedies. While adherence-adjusted would include completion of treatment that is gentamicin injection for 2 days and amoxicillin tablet for 7 days for full course completion of the treatment which is not included in this analysis. The manuscript is revised accordingly to clarify this. See page 11, lines 176-189.*

- It would be useful to show in a table format challenges identified in baseline, implementation strategies / interventions adopted, which strategies worked the most to improve effective coverage. at individual, HHs and community level

*Response: Thank you for the valid comments. We presented the strategies, details of the activities, and roles of stakeholders in tabular form (see Table 1, page 8)*

- **Discussion**: Clearly state the strategies and interventions that helped to remove key barriers and why some barriers continued to pursue such that coverage of newborns who took appropriate antibiotics was still low.

*Response: Thank you for the valid comments. We presented the strategies, details of the activities, and roles of stakeholders in tabular form (see Table 1, page 8) page 20, lines 323-334. In addition, the continued challenges for the treatment of PSBI are discussed. Page 22, lines 392-404*

- **Reference**: Please also add Mukhopadhyay R, Arora NK, Sharma PK, Dalpath S, Limbu P, Kataria G, et al. (2021) Lessons from implementation research on community management of Possible Serious Bacterial Infection (PSBI) in young infants (0-59 days), when the referral is not feasible in Palwal district of Haryana, India. PLoS ONE 16(7): e0252700. https://doi.org/10.1371/journal. pone.0252700

*Response: Thank you for sharing the article. We consulted it and* used the key findings in our discussion*. (Avan et al., 2021; Berhane et al., 2021; Mukhopadhyay et al., 2021).” Page 19, lines 313-320*
