# Supplementary material for: Effect of community-based newborn care implementation strategies on access to and effective coverage of possible serious bacterial infection (PSBI) treatment for sick young infants during COVID-19 pandemic
Source: PLoS One. 2024 Mar 25;19(3):e0300880. doi: 10.1371/journal.pone.0300880 (PMC10962833; doi:10.1371/journal.pone.0300880)
Supplement: Attachment — Submitted filename: Point-by-point response.docx [file pone.0300880.s006.docx]

**A point-by-point response to reviewers**

**PONE-D-23-12889R2**

**Effect of community-based newborn care implementation strategies on access to and effective coverage of possible serious bacterial infection (PSBI) treatment for sick young infants during COVID-19 pandemic**

Dear Editor,

*We, the authors, would like to thank the reviewers for their valuable comments. Our point-by-point responses to the reviewers and editor are below each comment. We also reviewed to ensure that this manuscript version conforms to the journal style.*

Reviewer Comments:

I have some reservation on the manuscripts:

Authors are trying to justify that the emergency adaptive PSBI treatment implementation strategies have changed a lot regarding treatment seeking and even time of seeking. Nonetheless, the authors did not statistically or plausibly justify the changes for the following reasons:

1. It was obvious that the overall use of essential service has declined in those year 2020 and 21, and expected to rise toward the end of 2022 as the health system started no reconsider the correction of the disruption of service at that time. What is your plausible (statistical and qualitative) evidence on this?

*Response: Thank you for the valid comments.* *Sure, in Ethiopia, the uptake of essential health services has been disrupted by the pandemic. But essential service delivery has been restored within a few months after the pandemic, particularly, in rural communities. The project abstracted longitudinal time series data from health post records and explored the trends in the monthly rate of PSBI treatment by HEWs pre-intervention, immediate, and after-project support effects. This interrupted time-series analysis showed the monthly rate increased by 4.1%, with an annual increase of 4.6%, higher than expected without the project's PSBI treatment strategies. This has been published elsewhere (Gizachew Tadele Tiruneh et al., 2024).*

*Still, we acknowledged that one of the limitations of this study is the lack of comparison group and pre-COVID-19 data to examine causality. As such, in this version, we included the following sentences in the limitations of the study. “The study, based on health post records, revealed a substantial increase in the monthly treatment rate by HEWs due to project support. However, limitations such as the absence of a comparison group and pre-COVID-19 data make it challenging to attribute outcomes solely to the intervention.” See on page 24, lines 418-421.*

2. There may be a lot of factors leading to the reported changes- the authors did not present adequate evidence to plausibly confirm that the changes due to their adaptive implementation strategies. Time, duration, logic, ratio, dose, and plausibility of the change not well convincing. Time- what if the difference in time of data collection (April) and June months is a reason for increase of decline of the PSBI? Other programs could also have contributed for the changes.

*Response: Thank you again for the comments. As described above, the time series data from health post records, controlling seasonal variations, revealed a substantial increase in the monthly treatment rate by HEWs due to project support. However , the pre-post only design nature of the study could not able to prove attribution of the strategies. In addition, as you righly said, time-varying unobserved confounders would bias the observed treatment effects. In this regard, however, as this an IR, we were contiously tracking other programs such as presence of NGO support for maternal and child healthas a facilitator in one woreda and conxtual factors such as civil conflict in another woreda were captured and made strategy adaptiaions to address these challenges to maitain high fidelity implementation of the straegies, published elsewhere (Tiruneh, Nigatu, Magge, & Hirschhorn, 2022). For this purpose, we used the IRLM framework to capture these emerging or persistent challenges and adapted strategies to articulate the mechanisms of action and link them to outcomes (Tiruneh, Nigatu, Magge, & Hirschhorn, 2022).*

3. The HEWs are practically overburdened with multiple tasks, and how do you justify such huge change?

***Response****: Sure, HEWs and other health workers in the primary care system are often reported being overburdened with multiple tasks. However, time motion and efficiency studies demonstrated that substantial time is spent waiting for clients (Arsenault et al., 2021; Tilahun et al., 2017).*

*We learned that performance of the HEW could be significantly improved if they are supported, mentored and supplied with necessary supplies as well as expanding service delivery modalities and demand creation activities. As such, the project implemented on-site coaching and training for HEWs, strengthened the supply chain and support system, and integrated the management of possible severe bacterial infection (PSBI) into the woreda health system work stream. This approach actively engaged HEWs, their supervisors, and WDAs in the delivery of PSBI treatment.*

4. The authors did not present other relevant domains of RE-AIM model to support the reported changes. example, what was the coverage of their intervention? and the adaption (individual and health system) dimensions?

***Response****: Thank you for the valid comments. The following paragraphs are included on line 145-159, pages 9-10. “The strategies were implemented effectively, ensuring the continued delivery of PSBI services while improving mothers' care-seeking behavior for their sick young infants. Technical support units comprised of iCCM trained personnel from under-five clinics of health centers, health center directors, and woreda health office child health officers were established. They provided on-site training and coaching to HEWs through supportive supervision, facilitated performance reviews, coordinated referrals, provided feedback to HEWs, identified implementation challenges, developed interventions in collaboration with stakeholders, and supported community sensitization efforts [16]. Monitoring data revealed that 84% of HEWs treated at least one sick young infant in the previous six months. Between April 2021 and May 2022, HEWs conducted 32,428 home visits, identifying 3,099 newborns, approximately three infants per health post per month. During the same period, HEWs assessed 1,335 severe young infant illness cases, representing an 85% coverage rate.*

*Implementation strategies were largely integrated into community-level healthcare worker workstreams, woreda reports, and the MOH’s annual planning and forecasting of iCCM/ PSBI commodities for 2023 and 2024.”*

Other comments

Is that true that the WDA in Ethiopia are responsible for educating neonatal danger signs- and what evidence can be presented to assure this statement in the background?

***Response****: Sure, according to the national guidance, WDAs with support from HEWs are expected to conduct home visits and educate mothers about maternal and newborn danger signs, identify sick newborns, and link to the facility (MOH, 2012). Accordingly, in settings where WDAs are well supported by the HEWs, they identified and referred mothers and newborns and linked them to HEP and/or health facilities. This resulted in improved uptake of institutional deliveries and newborn care practices such as clean cord care for newborns, thermal care for newborns, and immediate initiation of breastfeeding (Karim et al., 2018; Zufan Abera Damtew et al., 2018). However, in most cases, postpartum and newborn health care is less prioritized in the country (Tiruneh, Worku, Berhane, Betemariam, & Demissie, 2020) and WDAs have less engagement in newborn and child health (Ashebir, Medhanyie, Mulugeta, Persson, & Berhanu, 2020).*

**References**

Arsenault, C., Yakob, B., Tilahun, T., Nigatu, T. G., Dinsa, G., Woldie, M., . . . Kruk, M. E. (2021). Patient volume and quality of primary care in Ethiopia: findings from the routine health information system and the 2014 service provision assessment survey. *BMC Health Services Research, 21*(1), 485.

Ashebir, F., Medhanyie, A. A., Mulugeta, A., Persson, L. Å., & Berhanu, D. (2020). Women’s development group leaders’ promotion of maternal, neonatal and child health care in Ethiopia: a cross-sectional study. *Global health action, 13*(1), 1748845.

Gizachew Tadele Tiruneh, George Odwe, Alexandra Haake Kamberos, Kezia K’Oduol, Nebreed Fesseha, Zipporah Moraa, . . . Hirschhorn, L. R. (2024). Optimizing integration of community-based management of possible serious bacterial infection (PSBI) in young infants into primary healthcare systems in Ethiopia and Kenya: successes and challenges *BMC Health Services Research, Accepted*.

Karim, A. M., Fesseha Zemichael, N., Shigute, T., Emaway Altaye, D., Dagnew, S., Solomon, F., . . . Getachew, N. (2018). Effects of a community-based data for decision-making intervention on maternal and newborn health care practices in Ethiopia: a dose-response study. *BMC Pregnancy and Childbirth, 18*(1), 19-30.

MOH. (2012). *Community Based Newborn Care Implementation Guideline*. Addis Ababa, Ethiopia: Ministry of Health, Ethiopia.

Tilahun, H., Fekadu, B., Abdisa, H., Canavan, M., Linnander, E., Bradley, E. H., & Berman, P. (2017). Ethiopia’s health extension workers use of work time on duty: time and motion study. *Health Policy Plan, 32*(3), 320-328.

Tiruneh, G. T., Nigatu, T. G., Magge, H., & Hirschhorn, L. R. (2022). Using the Implementation Research Logic Model to design and implement community-based management of possible serious bacterial infection during COVID-19 pandemic in Ethiopia. *BMC Health Services Research, 22*(1), 1515. doi:10.1186/s12913-022-08945-9

Tiruneh, G. T., Worku, A., Berhane, Y., Betemariam, W., & Demissie, M. (2020). Determinants of postnatal care utilization in Ethiopia: a multilevel analysis. *BMC Pregnancy and Childbirth, 20*(1), 1-12.

Zufan Abera Damtew, Z. A. D., Ali Mehryar Karim, A. M. K., Chala Tesfaye Chekagn, C. T. C., Nebreed Fesseha Zemichael, N. F. Z., Bantalem Yihun, B. Y., & Barbara A. Willey, W. (2018). Correlates of the Women's Development Army strategy implementation strength with household reproductive, maternal, newborn and child healthcare practices: a cross-sectional study in four regions of Ethiopia.
